# Poly(ADP-ribose) polymerase inhibitor maintenance therapy in ovarian cancer: a single-center retrospective study

**DOI:** 10.3389/fonc.2026.1771241

**Published:** 2026-05-15

**Authors:** Sydney Pence, Kayla Dyson, Kristen Waters, John O. Elliott, Kellie Rath, Stuart Pierce, Amy Harper, Corinne Calo, Aine E Clements

**Affiliations:** 1 Department of Obstetrics and Gynecology, Ohio Health - Riverside Methodist Hospital, Columbus, OH, United States; 2 Ohio State University College of Medicine, Columbus, OH, United States; 3 Wright State University Boonshoft School of Medicine, Fairborn, OH, United States; 4 Research Institute, Ohio Health - Riverside Methodist Hospital, Columbus, OH, United States; 5 Department of Gynecology Oncology, Ohio Health - Riverside Methodist Hospital, Columbus, OH, United States

**Keywords:** fallopian tube cancer, homologous recombination, ovarian cancer, PARPi, peritoneal cancer

## Abstract

**Objective:**

To determine the effectiveness and tolerability of PARPi maintenance in a real-world patient population. Specifically, to assess duration of PARPi, the rate of secondary malignancies, rate of side effects, and survival.

**Methods:**

A retrospective review of patients with ovarian, fallopian tube, and primary peritoneal cancer at OhioHealth Riverside Methodist Hospital in Columbus, Ohio was performed. All patients diagnosed since March 2015 were reviewed for eligibility. Data was collected via EMR query and stored in REDCap. Charts were reviewed for multiple data points, including duration of PARPi use, side effects, secondary malignancies, survival, and recurrence. Data were reported descriptively with medians and ranges.

**Results:**

Ninety-seven patients met inclusion criteria. The median time patients were maintained on a PARPi was 9.8 months. Termination of patient’s PARPi was most often due to recurrence followed by side effects. Fatigue, anemia, and thrombocytopenia were the most common side effects reported. We report a PFS median of 12.5 months and a median OS of 40.6 months following first PARPi exposure. When stratified by homologous recombination deficiency (HRD) status, unadjusted analyses demonstrated longer PFS among HRD-positive patients compared to HRD-negative and HRD-unknown groups; however, this association was not consistently maintained after multivariable adjustment, and no statistically significant difference in OS was observed. There were two secondary malignancies identified in our study.

**Conclusions:**

We report similar survival compared to published trials despite shorter courses of therapy and higher rates of discontinuation due to side effects. Although HRD-positive status was associated with improved outcomes in unadjusted analyses, this effect was not sustained after adjustment for confounding variables, underscoring the complexity of these associations in real-world populations. This cohort study emphasizes the need to examine the real-world population for accurate patient guidance and counseling.

## Highlights

- PARPi may prolong PFS in a real-world cohort less than what is expected from trials
- Side effects may lead to higher rates of discontinuation in a real-world setting
- Patient characteristics should be evaluated to provide accurate counseling

## Introduction

Poly(ADP-ribose) polymerase inhibitors (PARPi) are used as maintenance therapy for ovarian cancer, following a complete or partial response to treatment, most often in the setting of homologous recombination deficiency (HRD) or Breast Cancer Associated 1 and 2 (BRCA 1 and 2) gene mutations (1, 2). PARPi work by inhibiting the ability of PARP to repair breaks in single strand DNA (ssDNA) (3). These ssDNA breaks are subsequently converted to double stranded DNA (dsDNA) breaks which can be repaired by homologous recombination (HR); however, in ovarian cancers that are HRD, cell death ensues (3). BRCA 1 and 2 genes have shown to have an essential role in accurate homologous recombination (4, 5). Thus, cancers associated with BRCA1 and 2 mutations have historically been the target of PARPi maintenance therapy. More recently, HRD assays have been approved as a biomarker for the use of PARPi due to similar effects on repair of dsDNA (3).

Currently olaparib, niraparib, and rucaparib are the PARPi approved for use. These agents were approved after being shown, in separate studies, to prolong progression free survival (PFS) (6–8). As trial data has matured, olaparib has since been shown to prolong overall survival (OS) (9). Despite these recent breakthroughs in ovarian cancer maintenance therapies, PARPi do have some undesirable side effects including thrombocytopenia, anemia, neutropenia, hypertension, and an increased risk of secondary malignancies, specifically myelodysplastic syndrome and acute myelogenous leukemia (6–12). The risk of secondary malignancies has been reported to be < 1% (11).

In the maintenance randomized studies of upfront treatment, PARPi was stopped after 2–3 years. Stopping treatment after 2–3 years is based on concern that there may be increased risks of side effects, particularly the risk of secondary malignancies, with prolonged use (13). However, it is possible that with continued use, remission may be prolonged further. Thus, the recommendation from the American Society of Clinical Oncology as well as the Society of Gynecologic Oncology state that a longer duration should be considered in patients deriving benefit from the PARPi, following discussion with patient regarding risks (1, 2).

There are relatively few studies evaluating PARPi safety and effectiveness outside of a trial setting. This data is important given the exclusivity of trial populations which can make its data difficult to broadly apply. Existing real-world analyses generally suggest that PARPi maintain meaningful PFS while also revealing important variations in outcomes and toxicity profiles (14–18). Despite these findings, gaps remain in understanding outcomes in a heterogenous real-world clinical practice. The objective of this study is to determine the effectiveness and tolerability of PARPi in a real-world patient population. Specifically, we aim to assess duration of PARPi, the rate of secondary malignancies, rate of side effects from PARPi maintenance therapy, and survival.

## Methods

### Study overview

A retrospective review of ovarian cancer patients at Ohio Health Riverside Methodist Hospital in Columbus, Ohio was performed from March 2015 until December 2023. The study was approved by the ethics committee at our institution (IRB # FY24-134).

### Patient eligibility

All patients with a diagnosis of ovarian, fallopian tube, or primary peritoneal cancer since March 2015, when our hospital electronic medical record was installed, were reviewed for eligibility. Patients were eligible for the study if they received care with OhioHealth Physician Group Gynecologic Oncology at Riverside Methodist Hospital, a large tertiary care center in Columbus, OH, and were diagnosed from March 1, 2015 until December 31, 2023. It was required that they were on a PARPi, including olaparib, niraparib, or rucaparib, for maintenance therapy after complete or partial response to chemotherapy. Patients were included in the study if the PARPi was prescribed after treatment of their primary ovarian cancer diagnosis or after recurrence. Patients were excluded if they had an active non ovarian malignancy at the start of their PARPi course.

### Data collection

Data were collected via the electronic medical record (EMR) and housed in the OhioHealth affiliated REDCap database. Charts were carefully examined by two independent reviewers to ensure accuracy in data collection.

Background demographics including age, race, ethnicity as well as preexisting health conditions including hypertension, chronic kidney disease, and diabetes were collected. Baseline data on the characteristics of their cancer were recorded including germline and somatic testing results, HRD status, histology, and stage at diagnosis. HRD status was determined through a review of patient charts, focusing on results from the Myriad HRD assay as well as germline and somatic BRCA mutation testing identified via genomic panels. Patients were classified as HRD-positive if they had a positive Myriad assay result or, in the absence of Myriad testing, a documented BRCA mutation. Those with a negative Myriad assay result were classified as HRD-negative. Patients without Myriad testing who either had no BRCA testing performed or had negative BRCA results were categorized as having unknown HRD status.

Data regarding PARPi treatment, including the medication used, whether bevacizumab was included in the regimen, as well as start and end dates were collected. For each treatment course, dosages were recorded alongside dose adjustments, the dates of the adjustments, and the reasons for them. Charts were reviewed for progressive or recurrent disease, as defined by physician documentation. Recurrence and progression data were collected in combination, defining recurrence in our data collection as patients with recurrent disease while on or following cessation of PARPi maintenance therapy after a previously reported period with no evidence of disease (NED) as well as patients with a progression of their disease while on or following cessation of PARPi maintenance therapy following a period of stable disease (SD). In data collection, specifications were made to recurrence while on PARPi maintenance therapy versus following the cessation of therapy. If recurrence occurred while on the PARPi, time on PARPi prior to recurrence reported in months to the nearest 0.5 was recorded. If recurrence occurred after cessation of PARPi therapy, time from cessation to diagnosis of recurrence was recorded, again in months to the nearest 0.5. Furthermore, secondary malignancies were recorded, again specifying occurrence while on PARPi therapy or after its cessation with the same method to record timing as it relates to PARPi use. PFS was assessed by determining time from start of PARPi therapy to recurrence, death, or last follow up. OS was determined from start of PARPi therapy to time of death or last follow up. Death was ascertained by chart review and patient status in the EMR. Date of last follow up was defined by the last clinical note in the patient’s EMR. Finally, side effects, disease status at end date, and reasons for discontinuation were collected per physician documentation.

### Data Analysis

Data was analyzed and reported descriptively using means, medians, ranges, and 95% confidence intervals (CI). Median PFS and OS were determined by Kaplan Meier curves and compared using the log-rank test. When survival data were stratified by HRD status, statistical analysis were run by the log-rank test. The shaded regions depict the 95% CIs and a dash line shows the median survival time. The number at risk over the study time frame are reported at 0, 20, 40 and 60 months.

Multivariate survival analysis was conducted for PFS and OS to examine HRD status (a positive test was set as the reference group) after controlling for medication type, concomitant bevacizumab use, stage, surgical debulking outcome and discontinuation of PARPi treatment due to side effects reporting hazard ratios (HR), 95% CI along with the p-value. All analyses were conducted in jamovi with R version 2.6.44.

## Results

### Demographics

Five hundred and fifty-nine patients with ovarian, fallopian tube, or primary peritoneal cancer were screened for this retrospective study, of which ninety-seven patients met inclusion criteria. Patients were excluded if they were not prescribed a PARP inhibitor (n = 404), had an active non-ovarian malignancy at the time of PARP inhibitor initiation (n = 5), had a diagnosis that predated the study period (n = 41), or did not continue care at our institution beyond the time of diagnosis (n = 12).

Demographic data of patients included in the study is outlined in **Table 1**. The median age of our patient population was 67 years old with 93.8% being Caucasian. The majority (50%) of patients were stage IIIC at the time of diagnosis with high grade serous as the most common histology. Sixty-nine of the ninety-seven patients (71.1%) had a complete initial debulking surgery. Fifty-three patients were HRD-positive (54.6%), and nine were HRD-negative (9.3%). Thirty-five patients (36.1%) were HRD-unknown. Of the HRD-positive patients, germline and/or somatic mutations in BRCA 1 and/or BRCA 2 were present in 30 patients (56.6% of HRD-positive patients, 30.9% of total cohort), as outlined in **Table 2**.

**Table 1:** Baseline patient demographics.

Baseline patient demographics		
Age	Median	Range
Age	67 (median)	43-88 (range)
Race	n	%
White/Caucasian	91	93.8
Black/African American	4	4.1
Asian	1	1
Declined to specify	1	1
Ethnicity	n	%
Not Hispanic or Latino	95	97.9
Hispanic or Latino	1	1
Declined to specify	1	1
Pre-existing disease	n	%
HTN	53	54.6
CKD	2	2.1
Diabetes	14	14.4

**Table 2:** Baseline cancer demographics.

Baseline cancer demographics					
**Stage at diagnosis**	**n**	**%**	Initial debulking outcome	n	%
I	2	2	Complete	69	71.1
II	11	11.3	Optimal	20	20.6
IIIA	3	3.1	Suboptimal	8	8.2
IIIB	6	6.2			
IIIC	49	50.5	**BRCA mutations**	**n**	**%**
IVA	8	8.2	Germline BRCA1	4	4.1
IVB	18	18.6	Germline BRCA2	8	8.2
			Somatic BRCA1	3	3.1
**Histology**	**n**	**%**	Somatic BRCA2	4	4.1
Clear cell carcinoma	1	1	Germline BRCA2 + Somatic BRCA2	6	6.2
Endometrioid carcinoma	3	3.1	Germline BRCA1 + Somatic BRCA1	4	4.1
Mucinous carcinoma	0	0	Germline BRCA1 + Somatic BRCA1 + Germline BRCA2	1	1.0
High grade serous carcinoma	77	79.4			
Low grade serous carcinoma	0	0	**HRD status**	**n**	**%**
Borderline	0	0	Positive	53	54.6
Transition cell or Brenner	1	1	Negative	9	9.3
Undifferentiated or Other	15	15.5	Unknown	35	36.1

### PARPi use

The ninety-seven patients included in this study were prescribed PARPi therapy. Of these patients, 49 were started on olaparib, 9 on olaparib plus bevacizumab, 31 on niraparib, 6 on niraparib plus bevacizumab, and 2 on rucaparib. 94 patients stopped their first PARPi course by the end of this study. This was most often due to recurrence (50 patients, 53.2%), followed by side effects (23 patients, 24.5%). Only 17 patients (18.1%) discontinued therapy due to completion of the two-year prescribed course. Four patients (4.3%) discontinued therapy due to other reasons (**Table 3**).

**Table 3:** Reasons for discontinuation of PARPi.

Reasons for discontinuation				
Reason	Primary PARPi		Secondary PARPi	
Recurrence	n	%	n	%
All, regardless of medication	50	53.2	11	50.0
Olaparib	21	22.3	5	22.7
Olaparib + bevacizumab	4	4.3	3	13.6
Niraparib	20	21.3	2	9.1
Niraparib + bevacizumab	4	4.3	0	0.0
Rucaparib	1	1.1	1	4.5
Side effects	n	%	n	%
All, regardless of medication	23	24.5	6	27.3
Olaparib	10	10.6	2	9.1
Olaparib + bevacizumab	3	3.2	1	4.5
Niraparib	7	7.4	3	13.6
Niraparib + bevacizumab	2	2.1	0	0.0
Rucaparib	1	1.1	0	0.0
2-year course completion	n	%	n	%
All, regardless of medication	17	18.1	2	9.1
Olaparib	15	16.0	0	0.0
Olaparib + bevacizumab	1	1.1	0	0.0
Niraparib	1	1.1	0	0.0
Niraparib + bevacizumab	0	0.0	2	9.1
Rucaparib	0	0.0	0	0.0
Other	n	%	n	%
All, regardless of medication	4	4.3	3	13.6
Olaparib	1	1.1	0	0.0
Olaparib + bevacizumab	1	1.1	2	9.1
Niraparib	2	2.1	0	0.0
Niraparib + bevacizumab	0	0.0	1	4.5
Rucaparib	0	0.0	0	0.0

Of the 97 original patients included in this study, 24 (24.7%) were switched to a second PARPi course. Among these 24 patients, 13 patients (54.2%) were switched from their first PARPi course due to inability to tolerate side effects, 8 patients (33.3%) were restarted on a PARPi following treatment of recurrent disease, 1 patient (4.2%) started a second course after two-year completion of their first course, and 2 patients (8.3%) switched for other reasons. Regarding treatment transitions, nine patients originally maintained on niraparib were switched to olaparib. The remaining fifteen patients were originally on olaparib. Eight were switched to niraparib, one was switched to rucaparib, and six were prescribed a second course of olaparib. By the end of the study, 22 of the 24 patients had discontinued their second PARPi course. The most common reason for discontinuation was disease recurrence, accounting for 11 patients (50.0%), followed by side effects in 6 patients (27.3%). Two patients (9.1%) completed their full two-year course, while 3 patients (13.6%) discontinued treatment for other reasons (**Table 3**).

Five patients were still on a first or second PARPi course at the end of the study. Excluding these patients, the median duration of PARPi use was 9.8 months (0.2-36.8 months). With combination of their first and second PARPi courses, 24 of the 97 patients in the study (24.7%) completed a two-year course of therapy.

### Rate of side effects

Fatigue and anemia were the most common side effects reported in patients during their initial PARPi course (**Table 4**). Side effects led to a dose reduction in 56 patients, which accounts for 57.3% of patients.

**Table 4:** Number of patients affected by each side effect with primary and secondary PARPi therapy.

PARPi side effects					
Side Effect and Treatment Group	Primary PARPi	Secondary PARPi	Side Effect adn Treatment Group	Primary PARPi	Secondary PARPi
Thrombocytopenia	n (% of subgroup)	n (% of subgroup)	Renal Insufficiency	n (% of subgroup)	n (% of subgroup)
All	24 (24.7)	5 (20.8)	All	13 (13.4)	3 (12.5)
Olaparib	6 (12.2)	3 (30.0)	Olaparib	11 (22.4)	0 (0)
Olaparib + bevacizumab	1 (11.1)	1 (20.0)	Olaparib + bevacizumab	1 (11.1)	0 (0)
Niraparib	11 (35.5)	0 (0)	Niraparib	1 (3.2)	3 (42.9)
Niraparib + bevacizumab	4 (66.7)	1 (100)	Niraparib + bevacizumab	0 (0)	0 (0)
Rucaparib	2 (100)	0 (0)	Rucaparib	0 (0)	0 (0)
Anemia	n (% of subgroup)	n (% of subgroup)	Fatigue	n (% of subgroup)	n (% of subgroup)
All	36 (37.1)	5 (20.8)	All	37 (38.1)	4 (16.7)
Olaparib	19 (38.8)	3 (30.0	Olaparib	23 (46.9)	1 (10.0)
Olaparib + bevacizumab	5 (55.6)	0 (0)	Olaparib + bevacizumab	2 (22.2)	2 (40.0)
Niraparib	8 (25.8)	2 (28.6)	Niraparib	9 (29.0)	1 (14.3)
Niraparib + bevacizumab	2 (33.3)	0 (0)	Niraparib + bevacizumab	3 (50.0)	0 (0)
Rucaparib	2 (100)	0 (0)	Rucaparib	0 (0)	0 (0)
Neutropenia	n (% of subgroup)	n (% of subgroup)	Nausea	n (% of subgroup)	n (% of subgroup)
All	12 (12.4)	0 (0)	All	27 (27.8)	4 (16.7)
Olaparib	2 (4.1)	0 (0)	Olaparib	17 (34.7)	0 (0)
Olaparib + bevacizumab	3 (33.3)	0 (0)	Olaparib + bevacizumab	3 (33.3)	2 (40.0)
Niraparib	5 (16.1)	0 (0)	Niraparib	6 (19.4)	2 (28.6)
Niraparib + bevacizumab	1 (16.7)	0 (0)	Niraparib + bevacizumab	1 (16.7)	0 (0)
Rucaparib	1 (50)	0 (0)	Rucaparib	0 (0)	0 (0)
Hypertension	n (% of subgroup)	n (% of subgroup)			
All	11 (11.3)	0 (0)			
Olaparib	6 (12.2)	0 (0)			
Olaparib + bevacizumab	1 (11.1)	0 (0)			
Niraparib	3 (9.7)	0 (0)			
Niraparib + bevacizumab	1 (16.7)	0 (0)			
Rucaparib	0 (0)	0 (0)			

In the patients prescribed a second PARPi course, the most common side effects were thrombocytopenia and anemia, followed by fatigue and nausea (**Table 4**). These side effects led to dose reductions in 12 of the 24 patients (60%).

### Rate of secondary malignancies

There were two (2.06%) secondary myelodysplastic syndromes identified in our study. One developed 14.5 months after discontinuation of niraparib. The other case occurred in a patient on olaparib who switched to niraparib secondary to side effects. She developed a myelodysplastic syndrome 8 months after discontinuation of therapy, which was 25 months after she switched agents. There were two additional patients with a second primary cancer diagnosed during their PARPi use. One was a colon cancer and the other, breast cancer. These were diagnosed at 19.5 months and 23 months of PARPi use, respectively.

### Survival

Seventy-three of the ninety-seven patients had ovarian cancer recurrence. Of the 73, 52 patients recurred while on their PARPi therapy. In these patients, the average time on PARPi to recurrence was 7.9 months (1 – 24.5 months). The remaining 21 patients who recurred did so following PARPi maintenance therapy discontinuation. The average time from discontinuation of therapy to recurrence in these patients was 7.21 months (1–23 months). Of these 21 patients, only two (9.5%) had completed a 2-year course of therapy prior to discontinuation.

Of the 24 patients prescribed a second PARPi course, 15 experienced ovarian cancer recurrence. Twelve patients were diagnosed with recurrence while on their PARPi therapy, with an average time to recurrence of 7.6 months (1.5–23 months). Three patients were diagnosed with recurrence following completion of PARPi therapy, with average time to recurrence after discontinuation of PARPi of 10.3 months (6.5–17 months). Only one of these three patients (33.3%) had completed a 2-year course of therapy.

**Figure 1:**
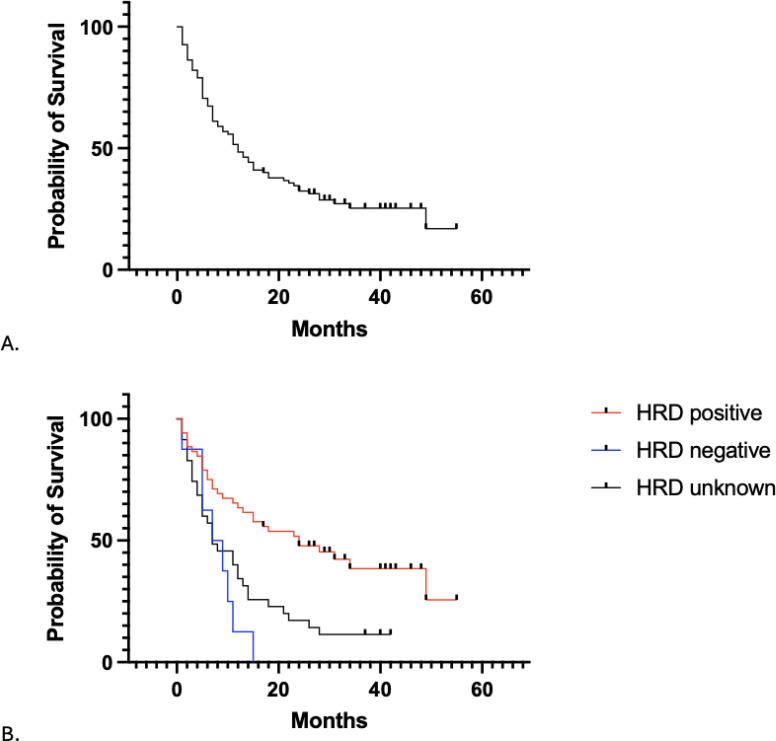
Progression free survival (months) **(A)** in all patients and **(B)** separated by HRD status.

Analysis of survival outcomes following the first PARPi exposure demonstrated a median PFS of 12.5 months (95% CI 8.2-18.5) (**Figure 1A**). When stratified by HRD status, the median PFS for HRD-positive, negative, and unknown patients were 24.6 months, 7.6 months, and 7.7 months, respectively (**Figure 1B**). There was a significant difference among these groups (p < 0.001). Patients who discontinued therapy due to side effects had a significantly lower likelihood of remaining progression-free compared to those who did not discontinue treatment (HR = 0.20, 95% CI 0.11-0.38, p<0.001). After controlling for medication type, concomitant bevacizumab use, stage, surgical debulking outcome and discontinuation of PARP treatment due to side effects, HRD-negative status was associated with improved PFS (HR = 3.24, 95% CI: 1.39 – 7.59, p = 0.007). However, this finding should be interpreted cautiously given the small sample size.

**Figure 2:**
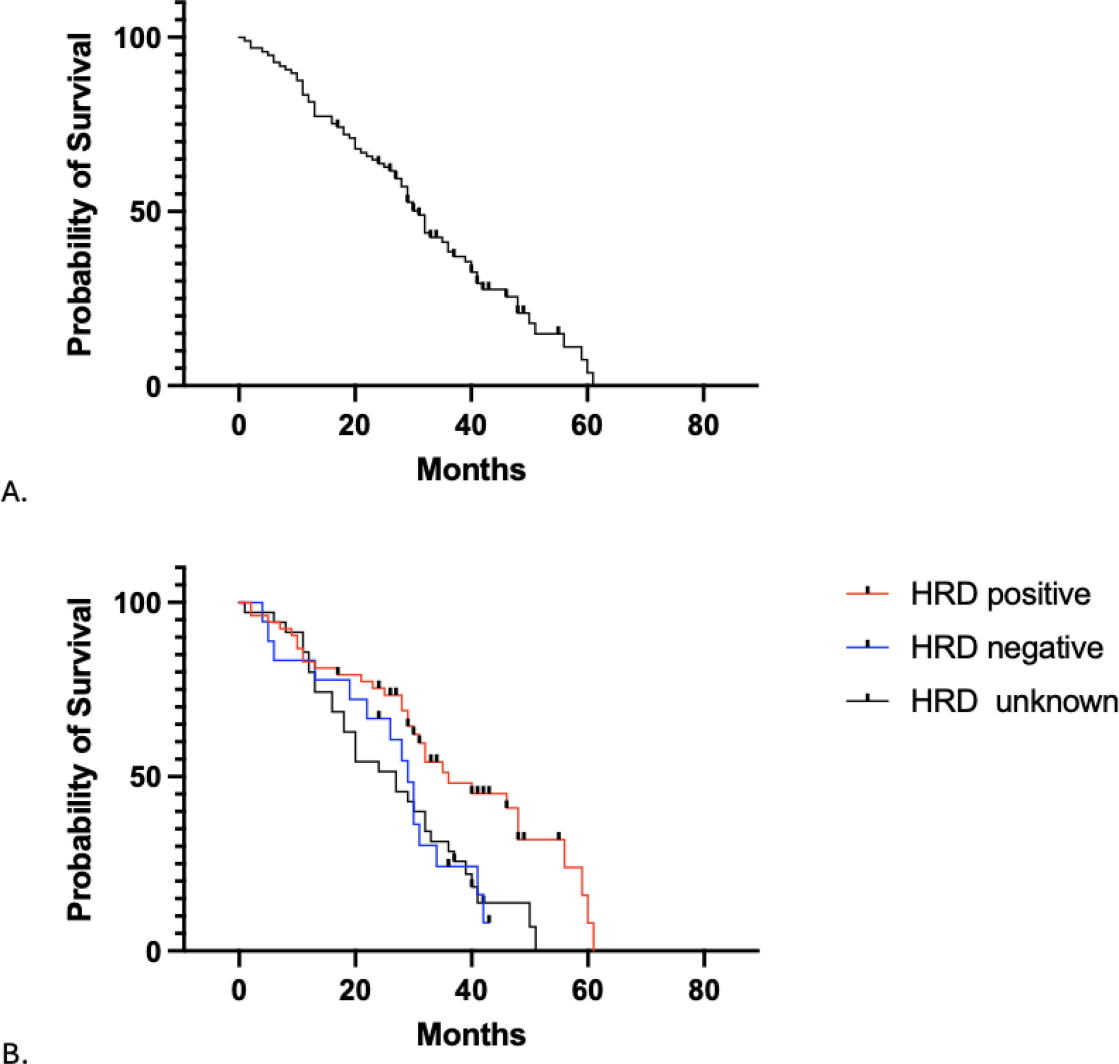
Overall survival (months) **(A)** in all patients and **(B)** separated by HRD status.

Median OS was 40.6 months (95% CI 30.7-NA) (**Figure 2A**). Median OS was 34.7 months for the HRD-positive cohort, 30.3 months for the HRD-negative cohort, and 41.3 months for HRD-unknown cohort (**Figure 2B**), with no statistically significant difference between groups (p = 0.054). As with PFS, patients who discontinued therapy due to side effects were significantly less likely to survive than those who did not (HR = 0.37, 95% CI 0.18-0.75, p=0.006). HRD-unknown status was associated with improved OS (HR = 2.50, 95% CI: 1.22 – 5.12, p = 0.013) after controlling for medication type, concomitant bevacizumab use, stage, surgical debulking outcome and discontinuation of PARP treatment due to side effects.

Median PFS and OS were also evaluated by treatment regimen. No differences were observed between olaparib vs olaparib plus bevacizumab or niraparib vs niraparib plus bevacizumab, although this analysis was limited by small sample size.

## Conclusions

This study gave insight into the use of PARPi maintenance therapy in a real-world patient population, including those with BRCA mutations, homologous recombination deficient tumors, and homologous recombination proficient tumors. Given this heterogenous population, it is difficult to directly compare to trial populations.

We showed that our patient population was maintained on PARPi therapy for a median of 9.8 months (0.2-36.8 months), with 24 patients (24.7%) completing at least 2 years of therapy. This analysis excluded five patients who were still receiving therapy at the end of the study time frame, which could underestimate treatment duration. In SOLO 1, all patients had a somatic or germline BRCA mutation and were treated with olaparib or placebo following treatment of newly diagnosed ovarian cancer. Of the patients treated with olaparib, 47% completed two years of therapy with the median duration of therapy being 24.6 months (19). Our population included patients with recurrent disease and had a small portion of patients with BRCA mutations but did demonstrate a shorter median duration of treatment in comparison. This was similarly demonstrated when comparing our data to the PAOLA trial which studied olaparib plus bevacizumab for maintenance treatment in patients following primary chemotherapy, regardless of HRD status. The treatment group in this trial was heterogenous, as was our cohort, and consisted of 47% HRD-positive, 36% HRD-negative, 17% HRD-unknown. The median duration of therapy was 17.3 months (10). Finally, the ATHENA-MONO is a placebo-controlled trial assessing rucaparib maintenance therapy following response to a primary treatment course. Patients were stratified based on BRCA status, with similar rates of BRCA mutations to our cohort at 21.3% of the those in the rucaparib group. In this trial a median treatment duration of 14.7 months was reported (20).

The most common reason for discontinuation was recurrence followed by side effects. Rates of discontinuation due to side effects in our study appeared to be higher than in trial populations, although this interpretation is limited by lack of adverse event grading in our data set. In our cohort, 23.7% of patients on their first PARPi maintenance course and 26.1% of patients on their second PARPi maintenance course ended treatment secondary to side effects. Dose reductions occurred in 57.3% of patients during first PARPi course and 60% of patients on their second PARPi course. The ENGOT-OV16/NOVA trial included patients with platinum—sensitive recurrent disease and were enrolled based on presence or absence of BRCA mutation. Patients without a BRCA mutation were further stratified based on HRD status. All subgroups were then assigned in a 2:1 ratio to receive niraparib maintenance therapy or placebo. This trial reported a lower rate of discontinuation of therapy for side effects (14.7%) and a higher rate of dose reductions (66.5%) compared to our cohort (21). These findings are similarly reported in the PRIMA trial, which randomized patients to receive niraparib maintenance therapy or placebo following response to primary chemotherapy. Of those who received niraparib, 50.7% had tumors with homologous-recombination deficiency. They reported lower rates of discontinuation of therapy for side effects (12%) and higher rates of dose reductions (70.9%) (6). The difference in our findings compared to trials may be secondary to variances in physician practice patterns or the health and compliance of patients in trial versus real world settings. Future studies should focus on management of adverse effects in real world patient cohorts.

In our patient population maintained on PARPi during their first course of therapy, we report a PFS median of 12.5 months and OS median of 40.6 months. When stratified by HRD status, PFS was 24.6 months, 7.6 months, and 7.7 months for HRD-positive, negative, and unknown, respectively. Median OS was 34.7 months for the HRD-positive cohort, 30.3 months for the HRD-negative cohort, and 41.3 months for HRD-unknown cohort. In review of the landmark trials median PFS is reported to range from 9.3 months to 37.2 months, with higher PFS rates being reported in patients who have BRCA mutations or are HRD-positive and lower rates in HRD-negative or unknown patients (6, 10, 20–22). While our observed median PFS is consistent with these reports, the apparent benefit in the HRD-positive cohort was not maintained after adjustment for covariates. This finding should be interpreted cautiously given the small sample size. Additionally, the PRIMA trial reports higher rates of OS when HRD-negative data was excluded compared to the overall population, suggesting an advantage in survival for HRD-positive patients (6). While our data did not reach a statistical significance, a similar trend was observed in our cohort.

Finally, multiple studies have evaluated the risk of secondary malignancies. A meta-analysis of 28 randomized control trials identified a risk of myelodysplastic syndrome or acute myeloid leukemia to be <1% (11). In our study, we report secondary myelodysplastic syndromes in 2.06% of patients maintained on PARPi therapy. Additionally, two patients did have solid tumors as a secondary malignancy which is not evaluated in other studies and was unlikely due to PARPi therapy. When looking solely at myelodysplastic syndrome or acute myeloid leukemia, our patients have similar rates of occurrence compared to other studies.

This study gives insight into the effectiveness and tolerability of PARPi in a real-world patient population. Several limitations should be considered when interpreting these findings. First, the small sample size limited our ability to perform robust statistical analyses. Additionally, the study population was derived from a single center and was predominantly Caucasian, which may restrict external validity. As a retrospective cohort study reliant on physician documentation, our analysis is also subject to inherent limitations in data collection. In particular, adverse effects were not consistently graded, which constrained more detailed evaluation and interpretation of toxicity outcomes.

With consideration of these limitations, we observed survival rates and trends comparable to those reported in randomized trials, even in the setting of shorter treatment durations and higher rates of discontinuation due to side effects. While unadjusted analyses suggested improved outcomes among HRD-positive patients, this association was not consistently maintained after multivariable adjustment, and no statistically significant difference in OS was observed. Nonetheless, our findings generally align with trends reported in clinical trials and highlight the complexity of translating these results into real-world populations. While further data is needed for a more comprehensive interpretation, this analysis may help inform patient selection for PARP inhibitor therapy and guide discussions regarding expected outcomes, particularly for patients who may not meet typical trial eligibility criteria.

## Data Availability

The raw data supporting the conclusions of this article will be made available by the authors, without undue reservation.
